# Enhanced waterlogging tolerance in barley by manipulation of expression of the N‐end rule pathway E3 ligase *PROTEOLYSIS6*

**DOI:** 10.1111/pbi.12334

**Published:** 2015-02-06

**Authors:** Guillermina M. Mendiondo, Daniel J. Gibbs, Miriam Szurman‐Zubrzycka, Arnd Korn, Julietta Marquez, Iwona Szarejko, Miroslaw Maluszynski, John King, Barry Axcell, Katherine Smart, Francoise Corbineau, Michael J. Holdsworth

**Affiliations:** ^1^ Division of Plant and Crop SciencesSchool of BiosciencesUniversity of NottinghamLoughboroughUK; ^2^ Department of GeneticsFaculty of Biology and Environmental ProtectionUniversity of SilesiaKatowicePoland; ^3^ School of Mathematical SciencesUniversity of NottinghamUniversity ParkNottinghamUK; ^4^ SABMiller plc. SABMiller HouseSurreyUK; ^5^ Seed Biology LaboratoryUMR 7622 CNRS‐UPMCSorbonne UniversitésUniversité Pierre et Marie Curie‐Paris 6ParisFrance; ^6^ Present address: School of BiosciencesUniversity of BirminghamEdgbastonB15 2TTUK

**Keywords:** N‐end rule, waterlogging, targeted proteolysis, ERFVIIs, PRT6

## Abstract

Increased tolerance of crops to low oxygen (hypoxia) during flooding is a key target for food security. In *Arabidopsis thaliana* (L.) Heynh., the N‐end rule pathway of targeted proteolysis controls plant responses to hypoxia by regulating the stability of group VII ethylene response factor (ERFVII) transcription factors, controlled by the oxidation status of amino terminal (Nt)‐cysteine (Cys). Here, we show that the barley (*Hordeum vulgare* L.) ERFVII BERF1 is a substrate of the N‐end rule pathway *in vitro*. Furthermore, we show that Nt‐Cys acts as a sensor for hypoxia *in vivo*, as the stability of the oxygen‐sensor reporter protein MCGGAIL‐GUS increased in waterlogged transgenic plants. Transgenic RNAi barley plants, with reduced expression of the N‐end rule pathway N‐recognin E3 ligase PROTEOLYSIS6 (HvPRT6), showed increased expression of hypoxia‐associated genes and altered seed germination phenotypes. In addition, in response to waterlogging, transgenic plants showed sustained biomass, enhanced yield, retention of chlorophyll, and enhanced induction of hypoxia‐related genes. *HvPRT6* RNAi plants also showed reduced chlorophyll degradation in response to continued darkness, often associated with waterlogged conditions. Barley Targeting Induced Local Lesions IN Genomes (TILLING) lines, containing mutant alleles of *HvPRT6*, also showed increased expression of hypoxia‐related genes and phenotypes similar to RNAi lines. We conclude that the N‐end rule pathway represents an important target for plant breeding to enhance tolerance to waterlogging in barley and other cereals.

## Introduction

Oxygen is required in plants for energy production through respiration, and low‐oxygen conditions (hypoxia) lead to dramatically changed metabolism to provide alternative sources of ATP (Banti *et al*., 2013). Hypoxic conditions can occur through changes in the environment surrounding the plant (e.g. flooding), through physical barriers imposed by plant anatomy or during developmental processes with high energy demands (Bailey‐Serres and Voesenek, 2008; Bailey‐Serres *et al*., 2012; Banti *et al*., 2013; Vashisht *et al*., 2011). During flooding events, plant roots can become waterlogged and aerial parts may become submerged in turbid water, leading to reduced capacity for respiration and photosynthesis. Prolonged exposure to hypoxic conditions leads ultimately to cell death.

There are an increasing number of flooding events worldwide, possibly as a result of climate change, and therefore, there is an imperative to provide crop types with increased capacity to tolerate low‐oxygen conditions (Bailey‐Serres *et al*., 2012; Voesenek and Bailey‐Serres, 2013). Whereas aquatic flowering plants can tolerate submergence, few cultivated crop species show low‐oxygen tolerance mechanisms. Semi‐aquatic rice (*Oryza sativa*) shows two opposite strategies associated with flooding evasion, quiescence or escape (Bailey‐Serres and Voesenek, 2008; Bailey‐Serres *et al*., 2012). A quiescence response is controlled by the *SUBMERGENCE1A* (*SUB1A*) locus and relies on a suppression of growth and induction of fermentative metabolism until the stress is removed (Bailey‐Serres *et al*., 2009). The *SUB1A* locus is not present in standard breeding lines, but was originally identified in submergence‐tolerant lowland rice land races from the Indian subcontinent, and is now used extensively in breeding programmes (Bailey‐Serres *et al*., 2009; Xu *et al*., 2006). In contrast to SUB1A‐induced quiescence, deep‐water rice varieties may escape flooding through increased internode elongation, controlled by the *SNORKEL1*(*SK1*) and *SK2* loci (Hattori *et al*., 2009). In both cases, the genes controlling these responses have been cloned and shown to encode representatives of the group VII ethylene response factor (ERF) transcription factor subfamily (Hattori *et al*., 2009; Xu *et al*., 2006). Quiescence and escape strategies have also been identified recently in two Rumex species from contrasting hydrological niches (van Veen *et al*., 2013).

Barley is comparatively more susceptible to waterlogging than other cereals. Flooding is a limiting factor for barley production in regions with high rainfall, and average yield can be reduced by up to 50% as a result of waterlogging (Setter and Waters, 2003). Although there is a low heritability for waterlogging tolerance in barley, resistance to this stress is an important objective of breeding efforts in high‐rainfall areas of the world. Leaf chlorosis is an early symptom of barley waterlogging, associated with reduced chlorophyll content, reduction in photosynthesis and senescence, although a differential response to waterlogging in barley varieties was linked to the pattern of aerenchyma formation (Pang *et al*., 2004; Zhou, 2011). It has been suggested that selecting genotypes with least reduction in photosynthetic rate or total chlorophyll content may facilitate identification of tolerant types (Pang *et al*., 2004). Quantitative trait loci (QTL) associated with tolerance to waterlogging in barley have been identified using combinations of leaf chlorosis, plant biomass production and plant death (Li *et al*., 2008, 2013; Mano and Takeda, 2012; Zhou, 2011; Zhou *et al*., 2012).

**Figure 1:**
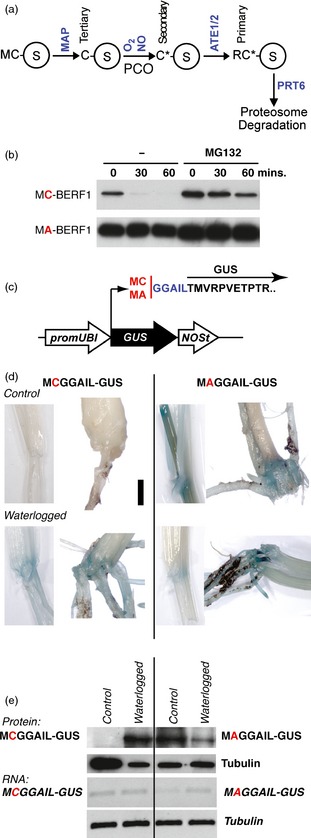
Amino terminal cysteine controls protein stability in barley. (a) The Cys‐Arg/N‐end rule pathway. Amino acids are indicated by single letters; C*, oxidized cysteine; MAP, methionine aminopeptidase; NO, nitric oxide; ATE, arginyl tRNA transferase; PRT6 E3, ligase PROTEOLYSIS6; and PCO, plant cysteine oxidase. Circled S indicates substrate proteins. Tertiary, secondary and primary Nt‐destabilizing residues are indicated. (b)  α‐HA Western blot analysis of the *in vitro* stability of HA‐tagged wild‐type and Ala^2^ variants of barley ERF1 (BERF1) in the absence or presence of MG132 following treatment with 100 μm cycloheximide at time point 0. Equal loading was confirmed by ponceau staining (data not shown). (c) Diagrammatical representation of the M(C/A)GGAIL‐GUS reporter construct used to analyse substrate stability *in vivo* in response to waterlogging. (d) Histochemical staining for GUS activity in transgenic barley leaf and root tissue following growth under well‐drained or waterlogged conditions for 15 days. In waterlogged conditions, the analysed tissue was submerged. (e) α‐GUS Western blot analysis of M(C/A)GGAIL‐GUS reporter and tubulin protein stability in response to waterlogging. Expression of RNA via semi‐quantitative rtPCR showing no change in GUS RNA expression in MC‐ or MA‐ constructs in response to waterlogging.

Two independent studies in arabidopsis (*Arabidopsis thaliana* (L.) Heynh.) demonstrated that the evolutionarily conserved N‐end rule pathway of targeted proteolysis is a key mechanism involved in the low‐oxygen response in plants (Gibbs *et al*., 2011; Licausi *et al*., 2011). The N‐end rule pathway relates the fate of a protein with the identity of its N‐terminal (Nt)‐residue (Bachmair *et al*., 1986; Gibbs *et al*., 2014a; Varshavsky, 2011). Proteins containing destabilizing Nt residues (N‐degrons) are ubiquitinated by specific E3 ligases (N‐recognins) and targeted for proteosomal degradation (Figure 1a). N‐degrons can be created by proteolytic, enzymatic or chemical modification of the Nt‐residue. In arabidopsis, PROTEOLYSIS6 (PRT6) is the N‐recognin for the arginine (Arg) branch of the N‐end rule pathway (Arg/N‐end rule) (Garzon *et al*., 2007; Graciet *et al*., 2009, 2010; Holman *et al*., 2009). Members of the group VII ERF (ERFVII) transcription factor subfamily were shown to be the substrates for this pathway, acting as oxygen sensors (Gibbs *et al*., 2011; Licausi *et al*., 2011). The arabidopsis subfamily includes five members, RELATED TO AP2 (RAP2) 2.12, 2.2, 2.3 AND HYPOXIA RESPONSIVE (HRE) 1 and 2 (Licausi *et al*., 2010; Nakano *et al*., 2006), each possessing a characteristic N‐terminal motif MCGGAII/L (Nakano *et al*., 2006). Constitutive removal of Nt‐methionine (Met) by Met aminopeptidase (MAP) (Frottin *et al*., 2006; Ross *et al*., 2005) reveals Nt‐Cys, a tertiary destabilizing residue (Varshavsky, 2011). An exposed N‐terminal Cys residue is vulnerable to oxidation, which permits subsequent arginylation by Arg‐tRNA protein transferases (ATEs; Figure 1a), followed by ubiquitination by N‐recognins recognizing the Arg destabilizing residue. Hence, Nt‐Cys acts as a sensor for oxygen (Hu *et al*., 2005; Lee *et al*., 2005) through the Cys‐Arg branch of the N‐end rule pathway. It was also shown that both nitric oxide (NO) and oxygen are required for conversion of Cys to its fully oxidized state (Cys‐sulphonic acid) and degradation of Nt‐Cys‐substrates and that plant‐specific enzymes (plant cysteine oxidases; PCOs) can contribute to this oxidation step using oxygen as a cofactor (Gibbs *et al*., 2014b; Hu *et al*., 2005; Weits *et al*., 2014). The rice SUB1A‐1 protein, though an ERFVII, was shown *in vitro* not to be a substrate of the N‐end rule pathway, suggesting that manipulation of components of the N‐end rule pathway to stabilize endogenous substrates may lead to enhanced tolerance to hypoxic environmental conditions. Two barley (*Hordeum vulgare* L.) ERFVIIs have previously been described and characterized; *HvRAF* was shown to be induced in barley seedlings by various treatments and conferred pathogen resistance and salt tolerance in transgenic arabidopsis (Jung *et al*., 2007), whereas *BERF1* is involved in fine‐tuning of expression of the Barley *knox3* (*Bkn3*) gene by ethylene (Osnato *et al*., 2010).

In this study, we demonstrate that barley possesses a functional Cys‐Arg/N‐end rule pathway and that Nt‐Cys, as part of the ERFVII consensus Nt‐sequence, acts as a sensor of waterlogging. We show that reduction in the expression or mutation of the barley N‐recognin E3 ligase *HvPRT6* gene leads to an increased tolerance to waterlogging, which is associated with changes in specific flooding‐related traits such as leaf chlorosis, chlorophyll content and biomass. Our work shows that the N‐end rule pathway and its proteolytic substrate proteins represent targets for the manipulation of resistance to waterlogging in barley.

## Results and discussion

### The barley ERFVII BERF1 is a substrate of the N‐end rule pathway, and amino terminal (Nt‐) Cys acts as a sensor for waterlogging status in barley

The ERFVIIs are characterized by the presence of the Nt‐conserved sequence MCGGAI(I/L) (Nakano *et al*., 2006). This sequence acts to sense oxygen and nitric oxide (NO) via the N‐end rule pathway of targeted proteolysis through the Nt‐Cys that is exposed following the action of MAPs (Figure 1a) (Gibbs *et al*., 2011, 2014b; Hu *et al*., 2005; Licausi *et al*., 2011). We searched the barley proteome (Mayer *et al*., 2012) initially to identify AP2‐domain proteins initiating MC‐ and then to define those with the consensus MCGGAI(I/L). There are 13 AP2‐domain‐containing proteins initiating MC‐ but only 7 sharing significant sequence similarity to the consensus Nt‐sequence of ERFVIIs (Figure S1, Table S1). Similarly, in rice, several ERFVIIs do not contain the full Nt‐consensus sequence (Gibbs *et al*., 2011). It is possible to distinguish subgroups with similarity to arabidopsis and wheat ERFVIIs (Figure S1b). We investigated whether barley ERFVIIs might be substrates of the N‐end rule pathway using an *in vitro* heterologous rabbit reticulocyte lysate assay (Gibbs *et al*., 2011; Lee *et al*., 2005), as components of the N‐end rule pathway are evolutionarily conserved (including enzyme activities of MAP, ATEs and UBR1 orthologue PRT6) (Graciet *et al*., 2010). We focused on the barley ERFVII family member BERF1, as it is most closely related in sequence to RAP2.12 in arabidopsis (Figure S1b), proposed to be the major oxygen sensing member of the subfamily (Licausi *et al*., 2011) and TaERF1 from wheat (Xu *et al*., 2007). We expressed C‐terminally haemagglutinin (HA)‐tagged BERF1 in this system and analysed the relative stability of native (Cys^2^) and mutated (Ala^2^) versions of the proteins in the presence and absence of the proteasome inhibitor MG132 following treatment with cycloheximide to block translation (Figure 1b). Whereas Cys^2^ protein was rapidly degraded in this system, and stabilized by MG132, conversion of Cys^2^ to Ala (a stabilizing residue; (Varshavsky, 2011)) greatly enhanced stability, indicating that BERF1 may be a substrate of the N‐end rule pathway *in vivo*.

To demonstrate that Nt‐Cys, as part of the barley ERFVII Nt‐consensus, can act as a sensor for hypoxia *in vivo* in transgenic barley plants, we analysed the stability of a modified β‐glucuronidase (GUS) reporter protein initiating with the consensus first 7 amino acids of the ERFVIIs, MCGGAIL‐ (or a mutated Ala^2^ version MAGGAIL‐) (Figure 1c). This reporter was previously used to demonstrate that Nt‐Cys senses NO in barley (Gibbs *et al*., 2014b). In this construct, transgene RNA expression is driven constitutively by the maize ubiquitin promoter (Bartlett *et al*., 2008). Under well‐drained normal growth conditions, no GUS staining could be observed in plants expressing the WT MCGGAIL‐GUS construction, whereas activity of the mutated Ala^2^ version (MAGGAIL‐GUS) could be visualized in the hypocotyl and primary roots. To impose waterlogging treatments, pots with seedlings (3–4 well‐developed leaves) were placed for 2 weeks in a plastic container filled with water. During the experiment, the level of water was maintained to ensure that the hypocotyl was covered. As a result of waterlogging, GUS activity could also be visualized in plants containing MCGGAIL‐GUS (Figure 1d) and Western blot analysis showed stabilization of GUS protein, which occurred in the absence of any changes in transgene mRNA levels (Figure 1e). These results demonstrate that Nt‐Cys, as part of the ERFVII consensus MCGGAIL, has the capacity to act as a sensor of waterlogging status. In arabidopsis, it was shown that HRE2 and RAP2.3 protein stability was enhanced by both hypoxia and submergence (Gibbs *et al*., 2011, 2014b), although no direct effect of root waterlogging on substrate stability has been shown. In conjunction with *in vitro* results using BERF1, our results suggest that barley ERFVIIs act as regulators of the barley response to waterlogging through their function as sensors of hypoxia via Nt‐Cys.

### Reducing expression of the barley N‐recognin E3 ligase *HvPROTEOLYSIS6* (*HvPRT6*) increases expression of hypoxia‐related genes

**Figure 2:**
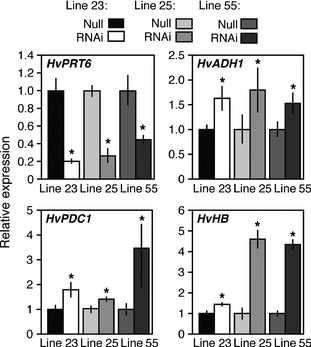
qRT‐PCR analysis of the influence of the *HvPRT6* RNAi construct on gene expression in independent barley homozygous RNAi lines compared to un‐transformed null segregant controls. Relative expression of *HvPRT6*,* HvADH1, HvHB*,* HvPDC1*. Transcript levels are shown relative to respective null segregants. In each case, error bars represent standard deviation of the mean. *P < 0.05.

The single‐copy gene *PROTEOLYSIS6* (*PRT6*) encodes the N‐recognin for the Arg branch of the N‐end rule pathway (Figure 1a) in arabidopsis (Garzon *et al*., 2007). We analysed the copy number of *PRT6* orthologues in other species using proteomes derived from sequenced plant genomes, including rice (Yu *et al*., 2002) (LOC_Os01g05500), *Brachypodium distachyon* (Vogel *et al*., 2010) (Bradi2g03180) and barley (Mayer *et al*., 2012) (MLOC_47469.1). In all cases, only a single protein was identified (Figure S2), and important domains for function, including the UBR box, RING finger domain and auto‐inhibitory domain, were found to be highly conserved across all species. Analysis of RNA levels for the barley *PRT6* orthologue (*HvPRT6*) indicates constitutive expression during growth and development (Figure S3). To test the role of HvPRT6 in regulating barley response to waterlogging, an RNAi approach was used. An RNAi construct was designed targeting a 600‐bp exon sequence corresponding to the region of the protein between the UBR box and RING domain features (Figure S2). Several lines derived from independent transformation events were generated, containing different copy numbers at single insertion sites for the RNAi construct (Table S2). Homozygous transgenic lines and associated null lines (nontransgenic but derived from the same transformation event) were initially analysed to observe if the introduced construct altered expression of the endogenous *HvPRT6* gene. In all cases, a large reduction in expression of *HvPRT6* RNA was observed (Figure 2). Genetic removal of the function of N‐end rule components in arabidopsis, including ATE and PRT6 (Figure 1a), leads to an increase in expression of genes associated with the core hypoxia response, including *ALCOHOL DEHYDROGENASE1* (*ADH1*), due to constitutive stabilization of ERFVIIs (Gibbs *et al*., 2011; Licausi *et al*., 2011; Mustroph *et al*., 2009). Analysis of leaf material of barley RNAi lines by quantitative (q) RT‐PCR revealed an increase in expression of the barley homologues of *ADH* (*HvADH1*), *HAEMOGLOBIN (HvHB)* and *PYRUVATE DECARBOXYLASE1* (*HvPDC1*) compared to untransformed controls. This indicates that reduction in HvPRT6 activity via RNAi silencing leads to an increased hypoxia‐associated gene expression. These increases were not as dramatic as seen in the arabidopsis *prt6* null mutant, which is likely due to the RNAi lines not completely abolishing expression of this gene.

### Reduced *HvPRT6* expression influences mature seed phenotypes

**Figure 3:**
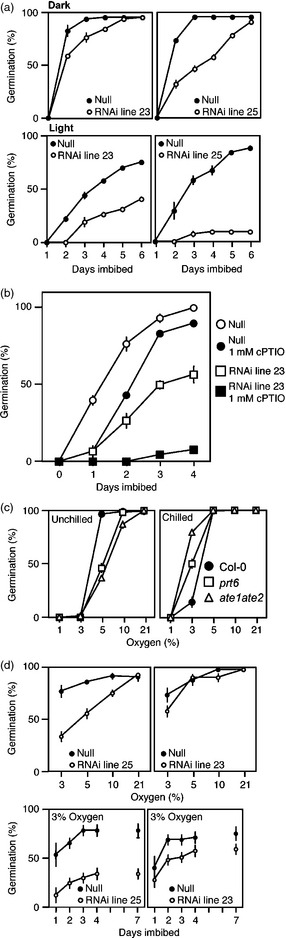
Reduced *HvPRT6* expression alters barley seed germination phenotypes. (a) Germination of two independent barley homozygous RNAi lines compared to un‐transformed null segregants under dark (top) or white light (bottom) conditions. (b) The influence of the NO scavenger cPTIO on germination in the light. (c) Germination of un‐chilled and chilled arabidopsis WT (Col‐0), *prt6* and *ate1ate2* seeds at 22 °C in continuous light and under reduced oxygen availability. (d) Germination of barley seeds at 21 °C under reduced oxygen availability following moist chilling. Final germination percentage at each oxygen concentration (top) and germination rate over 7 days at 3% oxygen (bottom) is shown. In each case, error bars represent standard deviation of the mean of three independent experiments.

The N‐end rule pathway controls many plant developmental and environmental responses (Gibbs *et al*., 2011, 2014a,b; Graciet *et al*., 2009; Holman *et al*., 2009; Licausi *et al*., 2011). In particular, it was shown that the pathway promotes seed germination and reduced abscisic acid (ABA) sensitivity by enhancing the degradation of ERFVIIs in response to NO (Gibbs *et al*., 2014b; Holman *et al*., 2009). Whereas in arabidopsis, light stimulates germination during imbibition, in barley, germination and removal of dormancy are enhanced in the dark (Gubler *et al*., 2008). We found that after‐ripened barley seeds completed germination rapidly in the dark, with germination being slightly delayed in the RNAi lines (Figure 3a). However, RNAi lines showed a severe reduction in germination in comparison with null controls in the light. These data strongly indicate that the N‐end rule pathway controls germination in cereals, and reduced germination in white light in the RNAi lines may result from increased stability of ERFVII substrates.

To link the Cys‐Arg branch of the N‐end rule pathway to the regulation of germination in barley, we analysed the influence on RNAi lines of the cell‐permeant NO scavenger 2‐(4‐carboxyphenyl)‐4,4,5,5‐tetramethylimidazoline‐1‐oxyl‐3‐oxide (cPTIO). Previously, we showed that the MCGGAIL‐GUS reporter was stabilized in barley embryos treated with cPTIO, suggesting that substrates of the Cys‐Arg/N‐end rule pathway act as sensors for NO in barley (Gibbs *et al*., 2014b). In addition, it was previously shown that NO can break dormancy in both barley and arabidopsis (Bethke *et al*., 2004), and arabidopsis dormancy is regulated specifically by ERFVII stability in response to NO (Gibbs *et al*., 2014b). We found that null segregant barley seeds showed a small reduction in germination speed in the presence of cPTIO in the light, whereas germination of RNAi seeds was almost completely inhibited (Figure 3b). These data indicate that in barley seeds, the N‐end rule pathway controls substrate stability and germination potential in a similar way to arabidopsis (Gibbs *et al*., 2014b; Holman *et al*., 2009). It has been suggested that the barley coleorhiza (an embryo‐derived tissue that covers the emerging root) may play a similar role to the arabidopsis endosperm (Barrero *et al*., 2009), implying that by analogy, the N‐end rule pathway may regulate barley germination in response to NO via crosstalk with ABA signalling in this tissue.

Arabidopsis *prt6* and *ate1ate2* mutant seeds that were moist chilled (to remove dormancy) showed enhanced germination under low oxygen compared to wild‐type (WT; accession Col‐0) seeds, which is presumed to result from constitutive expression of hypoxia‐survival genes in the mutants (Gibbs *et al*., 2011). However, we found that the response of un‐chilled arabidopsis seeds to hypoxia was very different. Whereas WT seeds showed almost complete germination even in 5% oxygen, seeds of *prt6* and *ate1ate2* showed a reduced ability to germinate (Figure 3c). Moist chilling of arabidopsis seeds has previously been shown to strongly repress ABA sensitivity and enhance GA sensitivity (Holman *et al*., 2009; Ogawa *et al*., 2003; Yamauchi *et al*., 2004). In barley, germination under hypoxia is strongly repressed by ABA and the surrounding hull (Benech‐Arnold *et al*., 2006; Bradford *et al*., 2008; Hoang *et al*., 2013; Mendiondo *et al*., 2010). We tested the germination sensitivity of two barley RNAi lines at different oxygen levels following moist chilling and found that in comparison with null segregants, the final germination levels and speed of germination were reduced at the lowest oxygen level (Figure 3d). These results, similar to what is observed in nonchilled arabidopsis N‐end rule mutants, indicate that stabilization of N‐end rule substrates reduces germination capacity under hypoxia, which may result from increased ABA sensitivity.

### Reduced *HvPRT6* expression increases tolerance to waterlogging

**Figure 4:**
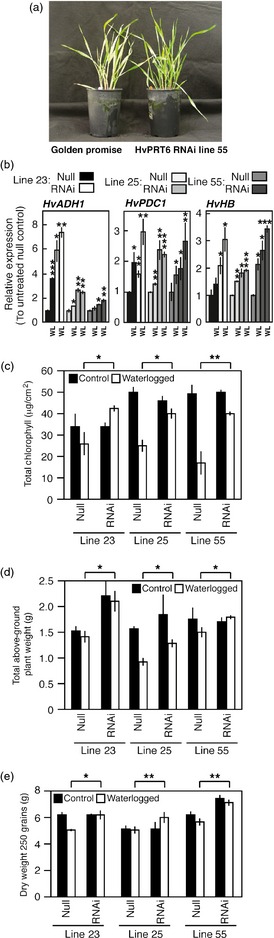
Reduced *HvPRT6* expression alters barley response to waterlogging. (a) Photograph of 25‐day‐old plants following 20 days of waterlogging, showing enhanced green leaf material in RNAi line 55 compared to WT. (b) Relative expression of hypoxia‐related genes measured by qRT‐PCR from healthy leaves of null and RNAi lines grown under well‐drained or waterlogged (WL) conditions. Transcript levels and significance are shown relative to well‐drained null segregant controls. (c) Total chlorophyll derived from leaves of plants grown under well‐drained or waterlogged conditions. (d) Total above‐ground plant weight following growth under well‐drained (control) or waterlogged conditions. (e) Total yield (dry weight of 250 grains) following growth under well‐drained (control) or waterlogged conditions. In each case, error bars represent standard deviation of the mean. *P < 0.05, **P < 0.01, ***P < 0.001.

Constitutive overexpression of native arabidopsis RAP2.12 (*35S:RAP2.12*) enhances survival in response to submergence and increases hypoxia‐related gene expression (Licausi *et al*., 2011). However, it was also shown in the same report that plant tolerance to flooding decreased in both *prt6* and *ate1ate2* mutants. This is in contrast to what is observed in rice lines containing the *SUB1A* locus, which have dramatically enhanced submergence tolerance (Bailey‐Serres *et al*., 2009). To determine the influence of reduced *HvPRT6* expression on tolerance to flooding, we examined the growth and physiological responses of barley *HvPRT6* RNAi lines to waterlogging. Following waterlogging treatment (20 days), several obvious differences in growth and development between nontransgenic and RNAi plants were observed. In comparison with null segregants, RNAi lines showed continued growth and maintenance of green leaves (Figure 4a). To quantify the responses to waterlogging, we analysed hypoxia‐related gene expression, chlorophyll levels, biomass and final yield. The expression of hypoxia‐related genes *HvADH1*,* HvHB* and *HvPDC1* in response to waterlogging was greater in RNAi lines than in null segregant controls (Figure 4b). One obvious difference in response to waterlogging was the retention of green coloration of transgenic leaves compared to controls, possibly as a result of enhanced maintenance of leaf chlorophyll content in response to the treatment. To investigate this, chlorophyll was extracted and quantified from leaves of null segregant and RNAi plants following waterlogging (Figure 4c). This analysis revealed a clear retention of chlorophyll in transgenic leaves compared to controls, suggesting that active photosynthesis continued in these lines despite waterlogging and also that leaf senescence was reduced. Both of these traits have previously been identified as hallmarks of waterlogging tolerance in barley, and we hypothesized that these factors may contribute to the relative enhanced growth of transgenics in excess water conditions. In agreement with this, analysis of total above‐ground biomass in response to waterlogging demonstrated that RNAi lines were relatively insensitive to the waterlogging treatment (Figure 4d), showing significantly less reduction in total plant weight in response to waterlogging compared to null segregants. As a consequence of this, transgenic lines also showed sustained yield under waterlogging conditions (Figure 4e). Collectively, our physiological analyses indicate that reducing *HvPRT6* levels has a positive effect on general growth and survival during waterlogging.

### Reduced *HvPRT6* expression enhances the maintenance of chlorophyll in leaves in continued darkness

**Figure 5:**
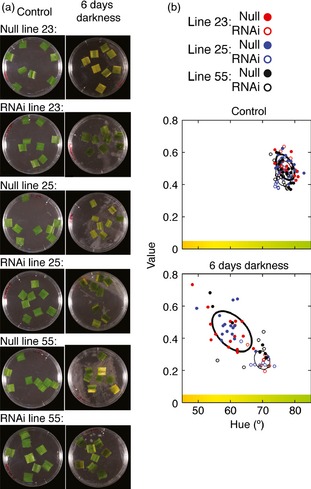
Reduced *HvPRT6* expression delays leaf senescence. (a) Photographs of leaf sections from RNAi lines and nontransgenic controls following treatment with darkness on liquid media for 6 days. (b) Quantification of colour change in leaf sections following transformation of images of leaf segments from RGB to HSV colour space. The 50% confidence regions are shown as ellipses (thick for null lines and thin for RNAi lines).

One symptom of plants exposed to prolonged foliar submergence is leaf senescence and chlorophyll degradation (Fukao *et al*., 2012; Vashisht *et al*., 2011). The effect of the *HvPRT6* RNAi construct on dark‐induced senescence was analysed using leaf segments incubated on one‐half‐strength Murashige and Skoog (MS) medium in darkness. Following 6 days of dark treatment, nontransgenic leaf segments appeared less green than those from RNAi lines (Figure 5a). To quantify this observation, we developed an image analysis approach using a transformation of images of the leaf segments from RGB colour space (red, green and blue) to HSV colour space (hue, saturation and value). Red, green and blue contributions were quantified, and from this, we calculated the hue of individual leaf patches (Figures S4, S5 and S6). As a result, we obtained a good measure of how green or yellow a leaf segment appears, allowing a quantification of colour change (Figure 5b). Using this procedure, it was possible to discriminate with statistical significance between null segregant and transgenic lines, showing that the colour of transgenic leaf sections did not alter in response to treatment as much as nontransgenic leaf sections. This implies a delay in leaf senescence in transgenics, similar to that previously observed in *SUB1A* in rice (Fukao *et al*., 2006, 2012). This suggests that stabilization of substrates in *HvPRT6* RNAi lines leads to enhanced tolerance to extended darkness, which may occur through repression of pathways activating leaf senescence and chlorophyll breakdown.

### Identification and analysis of Targeting Induced Local Lesions In Genomes (TILLING) lines containing mutant alleles of *HvPRT6*

**Figure 6:**
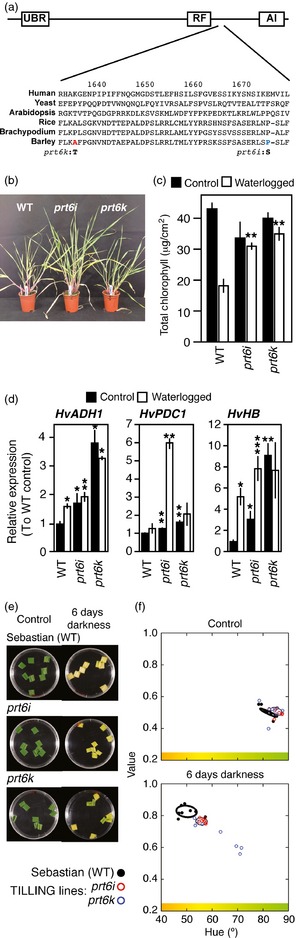
Phenotypes of two barley TILLING lines containing mutations in PRT6. (a) Positions of amino acid sequence changes in *prt6i* and *prt6k* TILLING lines. (b) Photograph of 20‐day‐old plants following 20 days of waterlogging, showing enhanced growth of TILLING lines compared to WT (Sebastian). (c) Total chlorophyll derived from leaves of plants grown under well‐drained or waterlogged conditions. (d) Expression of hypoxia‐related genes measured by qRT‐PCR from leaves of plants grown under well‐drained (control) or waterlogged conditions. (e) Photographs of leaf sections from RNAi lines and nontransgenic controls following treatment with darkness in 1/2MS liquid media for 6 days. (f) Quantification of colour change in leaf sections following transformation of images of leaf segments from RGB to HSV colour space. The 50% confidence regions are shown as ellipses (thick for Sebastian and thin for TILLING lines). *P < 0.05, **P < 0.01, ***P < 0.001.

To further investigate the role of HvPRT6 in barley waterlogging responses, we screened a‐mutagenized M2 population of barley variety Sebastian using the TILLING method (Kurowska *et al*., 2011; Weil, 2009) and identified two lines containing mis‐sense mutations in *HvPRT6*,* prt6i* (resulting in a proline to serine substitution; P1583S) and *prt6k* (resulting in an alanine to threonine substitution; A1543T) located between the Cys‐/His‐rich Ring domain and the C‐terminal auto‐inhibitory domain (Figure S2, Figure 6a). The *prt6k* mutation could be interpreted as conservative, as the arabidopsis PRT6 protein also contains threonine at this position, whereas the *prt6i* substitution changes a proline conserved in flowering plant PRT6 proteins. In both cases, mutant alleles resulted in decreased expression of *HvPRT6* RNA (Figure S7). Hypoxia‐related phenotypes and gene expression were analysed in the TILLING lines homozygous for *prt6* mutations and compared to WT (variety Sebastian). Growth was less affected by waterlogging in both lines compared to the WT (Figure 6b), chlorophyll levels were higher in TILLING lines following waterlogging (Figure 6c), and the expression of hypoxia‐related genes was increased in mutant lines relative to WT under both normal and waterlogged conditions (Figure 6d). Furthermore, dark‐induced leaf senescence was also delayed in the TILLING lines, as observed for RNAi lines (Figure 6e,f). Collectively, these data suggest that both TILLING lines have reduced HvPRT6 protein activity and corroborate our RNAi data to suggest that the N‐end rule pathway is a key regulator of waterlogging responses in barley. Further development of *HvPRT6* TILLING lines and analysis under field growth conditions may provide useful resources for breeders.

Our results indicate that manipulation of the N‐end rule pathway may provide approaches to increase tolerance to waterlogging, a stress that significantly affects barley productivity in areas with high rainfall. Collectively, our findings suggest that the ERFVIIs are N‐end rule substrates in barley that, alongside their known role in arabidopsis, implies that they also function as homeostatic sensors of hypoxia via the N‐end rule pathway in cereals. Transgenic *HvPRT6* RNAi lines had higher levels of anaerobic response gene expression, which translated into an increased tolerance to waterlogging, as indicated by stabilized growth and yield, greater chlorophyll retention and delayed dark‐induced leaf senescence under stress conditions relative to WT plants. Future examination of these lines should focus on analysing whether reducing N‐end rule activity positively or negatively affects responses to other stresses, such as drought (Fukao *et al*., 2011). Future research efforts will focus on targeted stabilization of individual barley N‐end rule substrates and assessment of their role in mediating the response to waterlogging *in vivo* in this important crop, and the development of nontransgenic TILLING alleles of *HvPRT6* to provide resources for breeders.

## Experimental procedures

### Plant material and growth conditions

The barley varieties Golden Promise (transformation) and Sebastian (TILLING) were used for all experiments. Unless otherwise stated, plants were grown under controlled conditions (15 °C/12 °C; 16‐h photoperiod; 80% RH, 500 μmol/m^2^/s metal halide lamps (HQI) supplemented with tungsten bulbs). Transgenic barley plants were generated using *Agrobacterium*‐mediated transformation of immature embryos from the variety Golden Promise (Bartlett *et al*., 2008). Regenerated transgenic plants were grown under the same conditions. Copy number and zygosity determination were carried out by IDna GENETICS (Norwich, UK). Waterlogging treatments were imposed as follows: well‐drained and waterlogging treatments were applied from developmental stage leaf 7 to leaf 10 (L7‐10). The leaf number at which waterlogging was applied was always measured on the main stem. Waterlogging duration was 15 or 20 days as indicated (de San Celedonio *et al*., 2014). Crop phenology was determined following the decimal code (Zadoks *et al*., 1974). To observe the effect of darkness on leaf senescence, fully expanded uppermost leaves were cut into 1‐cm^2^ sections and floated on one‐half‐strength MS liquid medium in the dark at 22 °C for up to 6 d in sealed Petri dishes (Fukao *et al*., 2012). Chlorophyll content was determined in triplicate samples by extraction and assay in 80% acetone (Porra *et al*., 1989). Histochemical staining for GUS activity was carried out as previously described (Gibbs *et al*., 2014b). Arabidopsis accessions and mutants were the same as those described previously (Gibbs *et al*., 2011, 2014b).

### Sequence identification and analysis

Basic local alignment search tool (BLAST) (Altschul *et al*., 1990) analysis was carried out to identify the barley orthologue of PRT6. CLUSTALW as part of the MacVector program (MacVector, Cambridge, UK) was used for multiple sequence alignments and to identify barley ERFVIIs from the predicted proteome derived from the barley genome sequence (Mayer *et al*., 2012). For RNAi silencing, a 530‐bp *HvPRT6* cDNA fragment (amplified using primers HvPRT6F and HvPRT6R; Table S3) was cloned into the pCR8GW donor vector (Invitrogen, Paisley, UK) and then into the RNAi destination vector pBract207 (Bartlett *et al*., 2008).

### RNA expression analysis

Total RNA was isolated from young leaf or root tissue as described previously (Mendiondo *et al*., 2014). Gene‐specific oligonucleotide primers (Table S3) were used in quantitative (q) RT‐PCR with SYBR‐green Sensimix (Bioline, London, UK), in a Roche LightCycler 480 apparatus (Roche/Applied Biosystems, Welwyn Garden City, UK) as specified by the manufacturer. The relative number of copies obtained for each transcript was normalized against *HvELF1* and *HvTubulin* transcript values for each sample as an internal reference (Jarosova and Kundu, 2010; Nicot *et al*., 2005).

### *In vitro* analysis of protein stability

The entire open reading frame of BERF1 was cloned from cDNA derived from leaf tissue and was directionally cloned into the modified pTNT3xHA vector to produce C‐terminal HA‐tagged fusions either as N‐terminally initiating MC or MA derivatives, and *in vitro* assays were carried out using rabbit reticulocyte lysate (Gibbs *et al*., 2011) but with the addition of 100 μm cycloheximide at the 0‐min time point to block mRNA translation.

### Western analysis of proteins

Western blots were carried out as previously described (Gibbs *et al*., 2011, 2014b). Anti‐HA antibodies were obtained from Sigma, and anti‐GUS antibodies were obtained from Invitrogen. Barley leaf samples (100 mg) were ground to powder with a mortar and pestle in liquid nitrogen, and protein extraction was performed (NucleoSpin^®^ RNA/Protein extraction kit, Macherey‐Nagel, Düren‐Germany). Total protein content in the samples was quantified by Bradford protocol with a spectrophotometer (Thermo Scientific Multiskan Ascent, Waltham, MA) against a BSA standard curve. For anti‐GUS Western blots, in each lane, 75 μg of total protein were loaded plus 5 μL of cracking buffer (0.5 m Tris–HCL, pH 6.8; 10% glycerol; 1% SDS; 5% β‐mercaptoethanol; 2 mg bromophenol blue) and water to a final volume of 20 μL. After 0.1% SDS–16.5% PAGE, proteins were electroblotted onto a nitrocellulose membrane. SeeBlue Plus2 Pre‐Stained Standard Marker (Novex, Paisley, UK) was loaded as a reference for protein size. Membranes were reversibly stained with Ponceau S Red to check equal loading and protein integrity.

### Seed germination assays

Heads were harvested at maturity, dried for 7 days and threshed by hand to prevent damage to the husk. For each assay, triplicates of thirty grains were placed in Petri dishes containing two layers of Whatman No. 1 filter paper (GE Healthcare Life Sciences, Little Chalfont, UK) and 4 mL sterile water. Dishes were sealed with Micropore tape (3 m) and incubated at 10 °C in the dark or white light. Germinated caryopses, defined by the emergence of coleorhizae beyond the seed coats, were scored every 24 h over 7 days and removed from the dishes. Germination under atmospheres with different controlled oxygen tensions was performed at 21 °C in darkness for barley and 22 °C for arabidopsis as described previously (Côme and Tissaoui, 1968). Gas mixtures containing from 1 to 21% oxygen were obtained through capillary tubes connected to sources of compressed air and nitrogen. The gaseous atmospheres were passed continuously through germination chambers at a constant flow rate (4.0 lh^1^). Oxygen tensions were measured daily using a Servomex analyser (type 570A; Servomex, Crowborough, UK).

### Quantification of leaf section colour

Images of leaf patches were acquired with Canon EOS 5D camera at a resolution of 27 pixels/mm. The size and shape differed between patches. For that reason and because the object could not be readily identified automatically, it was necessary to select sections of each patch for statistical analysis. Manual selection of sections (square or rectangular with an area of approximately 0.3 mm^2^) of leaves was carried out using the image analysis software Fiji (Schindelin *et al*., 2012). Subsequently, red, green and blue (RGB) colour channels were analysed in each section with a script written in Fiji's macro programming language (data S1; Figures S4, S5 and S6). Measurements of each region of interest included position within the micrograph, area in square pixels, mean brightness, median brightness and standard deviation of brightness. The mean RGB values of each leaf patch were converted to hue, saturation and value (HSV) using R and RStudio (RDevelopmentCoreTeam, 2012; RStudio, 2012). The formula used to calculate colour hues of yellow leaf patches is 60 degrees × ((G–B)/(R–B) mod 6) (mod refers to the modulo operation, and this formula is a special case for R > G > B and applicable for yellow leaf sections only). For green leaf sections, where G > R > B, the corresponding expression is hue = 60 degrees × ((B–R)/(G–B) + 2). The selection of the correct formula is carried out automatically within the built‐in R function ‘rgb2hsv’. The returned value is an arc length along a regular hexagon with total circumference of one. By multiplying by 360 degrees, an approximation of an angular coordinate is obtained. The structure of the hexagon is still recognizable in the full colour map as shown in Figures S5, S6 and 7, where 0 degrees corresponds to red, 60 to yellow, 120 to green, 180 to turquois, 240 to blue, 300 to magenta and 360 to red. These points correspond to six of the eight corners of the colour cube (taking R, G and B as Cartesian coordinates x, y and z, respectively). The remaining two corners correspond to black and white (which in HSV colour space involves the additional coordinates saturation and value). The 50% confidence regions (ellipses) shown here were obtained with the bivariate boxplot command bv.boxplot of the package asbio for R, implementing a previously described method (Goldberg and Iglewicz, 1992) and a biweight estimator function (Everitt, 2005).

### Identification of barley TILLING lines containing mutant alleles of *HvPRT6*

TILLING lines containing mutant alleles of *HvPRT6* were developed from the *Hor*TILLUS (*Hordeum vulgare*‐TILLING‐University of Silesia) population of spring barley cultivar ‘Sebastian’ created in Department of Genetics, University of Silesia, after double treatment of seeds with sodium azide (NaN_3_) and N‐nitroso‐N‐methylurea (MNU). Two different treatments were applied to two batches of seeds: 1.5 mM NaN_3_/3 h–6 h iig–0.75 mm MNU/3 h and 1.5 mm NaN_3_/3 h–6 h iig–0.5 mm MNU/3 h (iig—interincubation germination period). Mutation screening was performed on DNA isolated from 6,144 M_2_ *Hor*TILLUS plants. Eleven mutations were found in the *HvPRT6* gene, giving a mutation density of 1mut/605 base pairs. Two of these were chosen (*prt6i*,* prt6k*) that led to amino acid substitutions, theoretically resulting in an unaffected full‐length protein. For mutation identification, PCR was carried out using genomic DNA from eightfold DNA pools using the primers: forward (F): 5′‐(IRD700)‐ TGTCATGATCGATATTTGTTTTCC‐3′; reverse (R): 5′‐(IRD800)‐ TCGCTTAGTAGCATCCAAAAGA‐3′ that amplify a 695‐bp exon region of *HvPRT6* (Figure S2). For heteroduplex formation, samples were incubated at 95 °C for 3 min, and then, slow renaturation was performed in 70 °C per 20 s × 70 cycles (−0.3 °C/cycle). Heteroduplexes were cleaved with Celery Juice Extract as previously described (Till *et al*., 2006), and DNA fragments were visualized on 6% denaturing polyacrylamide gels. DNA from each individual from positive bulks was then analysed separately to identify single plants carrying mutations within the TILLING region. The DNA sequence of analysed fragments from plants carrying potential mutations in *HvPRT6* gene was obtained to confirm the presence of mutation and zygosity state. Seeds from M_2_ plants carrying mutations in *HvPRT6* gene were used for developing homozygous TILLING lines.

## Supporting information


**Figure S1** A. Alignment of the predicted protein sequences of Group VII ERFs from *Arabidopsis thaliana* and *Hordeum vulgare* and wheat TaERF1.
**Figure S2** Alignment of the predicted Barley PRT6 sequence with sequelogues from Human (*Homo sapiens*), yeast (*Saccharomyces cerevicieae*) and *Arabidopsis thaliana*.
**Figure S3** Expression of the barley PRT6 sequelogue during normal growth and development.
**Figure S4** Image analysis of a green and a yellow leaf patch (1 cm2) for quantification of greenness with hue in degrees.
**Figure S5** Hue histograms of individual pixels of a green and a yellow leaf patch.
**Figure S6** HSV colour space for visualisation of hue and value. Circles are filled with colours specified by their position within the plane of hue and value at maximum saturation of one.
**Figure S7** qRT‐PCR analysis of the influence of the *HvPRT6* mutant alleles *prt6k* and *prt6i* on *HvPRT6* gene expression relative to the WT (Sebastian).

**Table S1** Genome location of barley ERFVIIs (XLSX file).

**Table S2** Transgene copy number in RNAi transgenic lines (XLSX file).

**Table S3** List of oligonucleotides used in QrtPCR analyses (XLSX file).

**Data S1** ImageJ plug‐in software for analysis of leaf colour (IJM file).
