# Supplementary material for: Enhanced waterlogging tolerance in barley by manipulation of expression of the N‐end rule pathway E3 ligase PROTEOLYSIS6
Source: Plant Biotechnol J. 2015 Feb 6;14(1):40–50. doi: 10.1111/pbi.12334 (PMC5098238; doi:10.1111/pbi.12334)
Supplement: Supplementary file 1 — Figure S1 A. Alignment of the predicted protein sequences of Group VII ERFs from Arabidopsis thaliana and Hordeum vulgare and wheat TaERF1. Figure S2 Alignment of the predicted Barley PRT6 sequence with sequelogues from Human (Homo sapiens), yeast (Saccharomyces cerevicieae) and Arabidopsis thaliana. Figure S3 Expression of the barley PRT6 sequelogue during normal growth and development. Figure S4 Image analysis of a green and a yellow leaf patch (1 cm2) for quantification of greenness with hue in degrees. Figure S5 Hue histograms of individual pixels of a green and a yellow leaf patch. Figure S6 HSV colour space for visualisation of hue and value. Circles are filled with colours specified by their position within the plane of hue and value at maximum saturation of one. Figure S7 qRT‐PCR analysis of the influence of the HvPRT6 mutant alleles prt6k and prt6i on HvPRT6 gene expression relative to the WT (Sebastian). [file PBI-14-40-s004.pdf]

**Supplementary Figure 1**:

**A.** Alignment of the predicted protein sequences of Group VII ERFs from *Arabidopsis thaliana* and *Hordeum vulgare* and wheat TaERF1. Functional domains are highlighted according to Nakano *et al.* Plant Physiology (2006) 140 no. 2: 411-432. See **Supplementary Table 1** for more information about barely ERFVIIs.

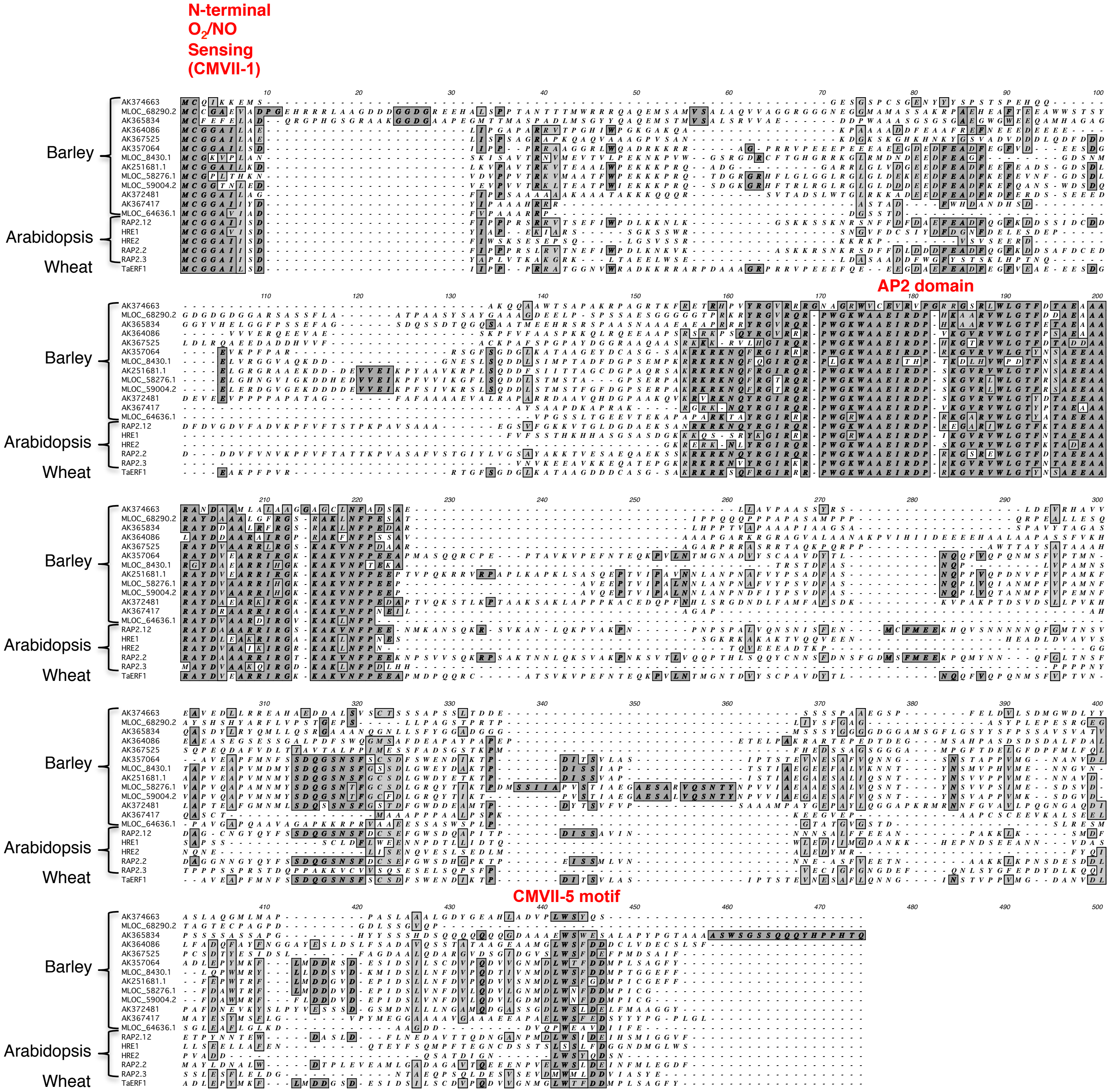


**B.** Neighbour joining tree showing the relationship between barley, wheat (TaERF1) and arabidopsis (RAP2.12, RPA2.2, RAP2.3, HRE1, HRE2) ERFVIIs.

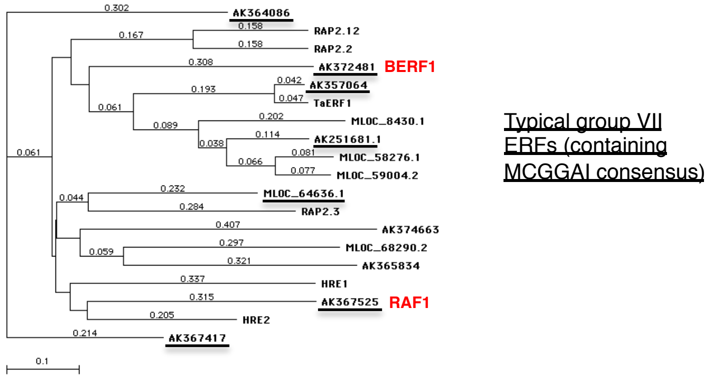


Conserved regions as described in Nakano, T., Suzuki, K., Fujimura, T. and Shinshi, H. (2006) Genome-wide analysis of the ERF gene family in Arabidopsis and rice. *Plant Physiol.* **140**, 411-432.

## Supplementary Figure 2

Alignment of the predicted Barley PRT6 sequence with sequelogues from Human (*Homo sapiens*), yeast (*Saccharomyces cerevicieae*) and *Arabidopsis thaliana*.

The positions of important protein domains and the region used to generate RNAi and TILLING lines are highlighted.

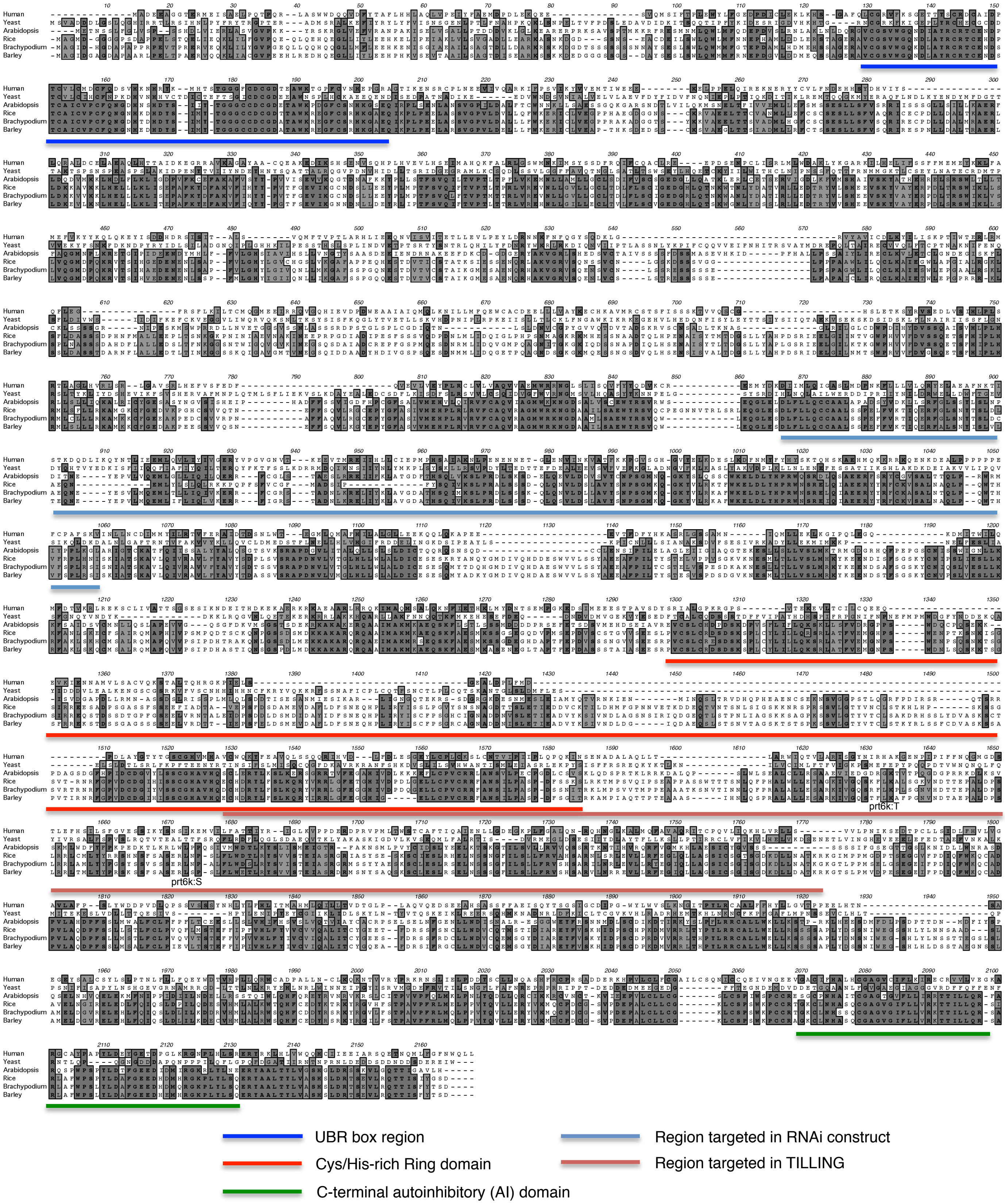

**Supplementary Figure 3**: Expression of the barley PRT6 sequelogue during normal growth and development. Data from the Barley eFP browser at bar.utoronto.ca, using the contig 7757.

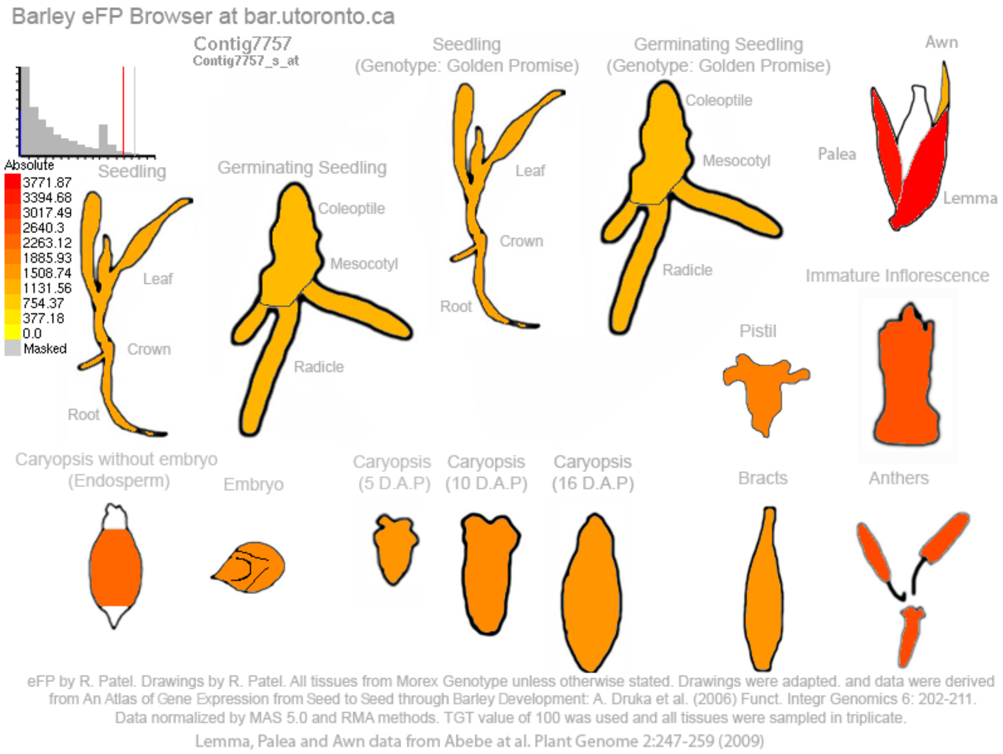

**Supplementary Figure 4.** Image analysis of a green and a yellow leaf patch (1cm$^2$) for quantification of greenness with hue in degrees. Regions of interest in colour micrographs (first row). Histograms of brightness values of individual pixels for the colour channels red, green, and blue (RGB, second row). Histogram of hue in degrees of individual pixels (third row). The thick black line denotes the mean hue of the image. Only a subsection (40…100 degrees) of the entire possible range of hue (0…360 degrees) is shown, see also **supplementary figures 5 & 6**.

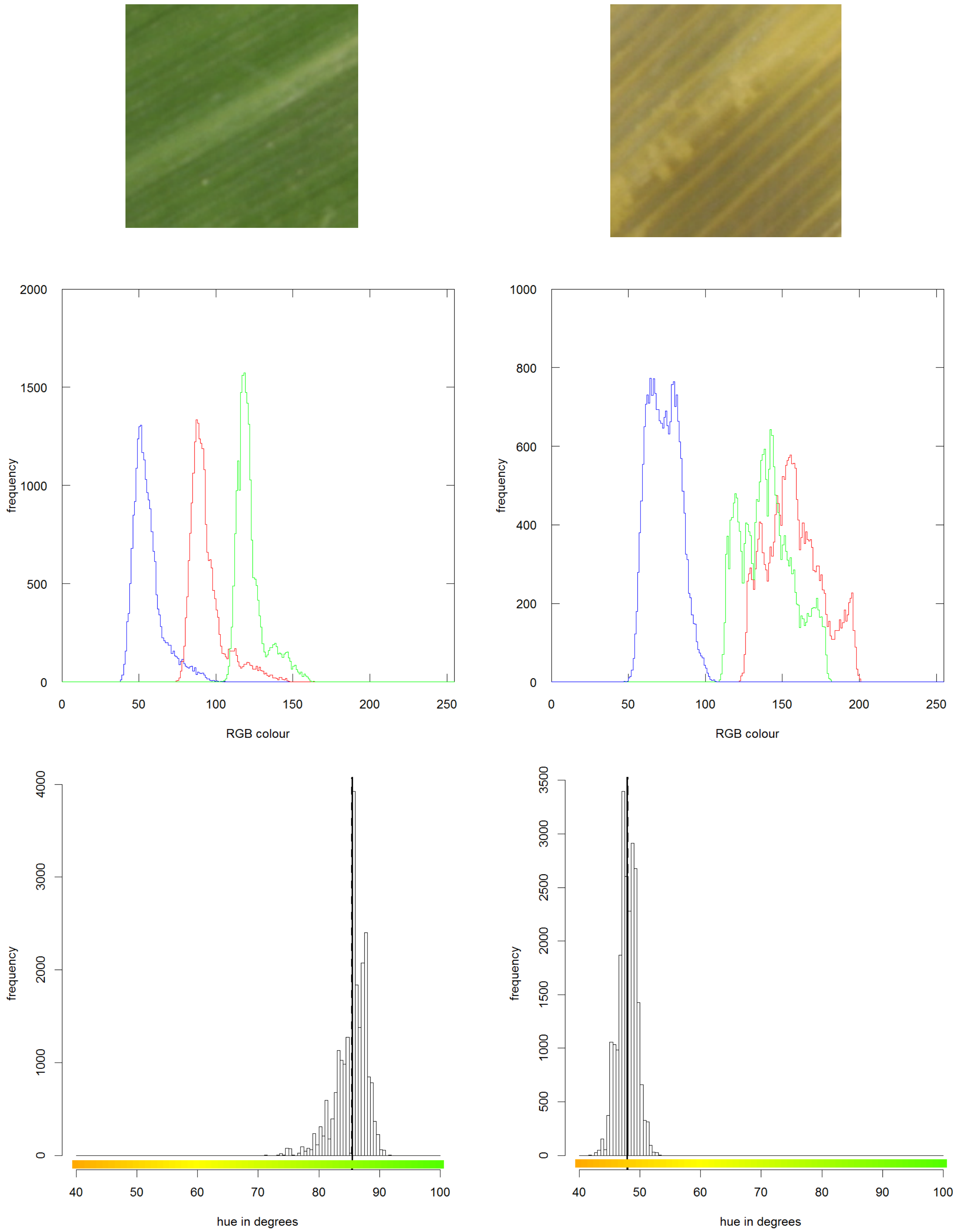

**Supplementary Figure 5**. Hue histograms of individual pixels of a green and a yellow leaf patch. The spread is much smaller than the spread in individual colour channels (see SOM Figure 1, second row).

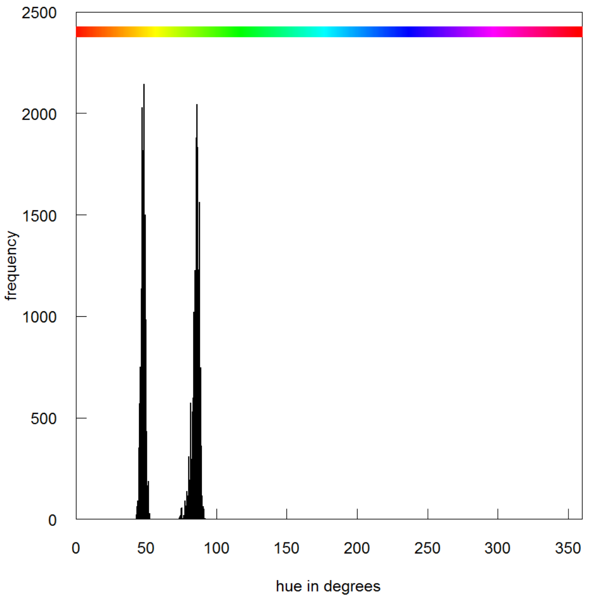


**Supplementary Figure 6**. HSV colour space for visualisation of hue and value. Circles are filled with colours specified by their position within the plane of hue and value at maximum saturation of one. The plot shows only a subset which is relevant for the colours of leaf patches analysed in this work (including green and yellow).

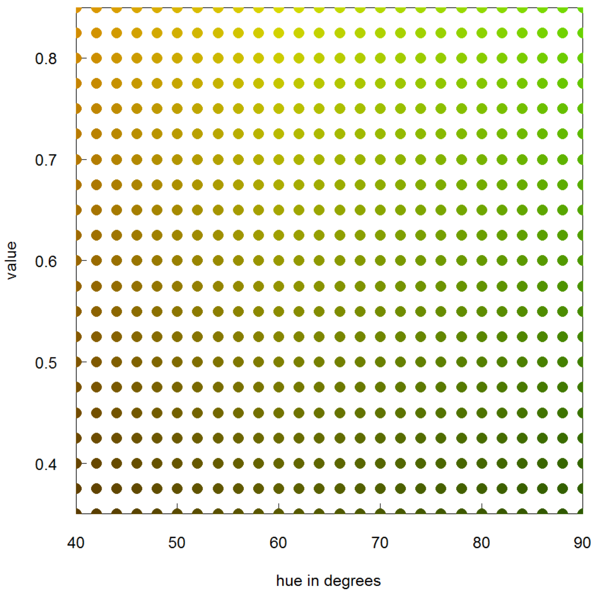

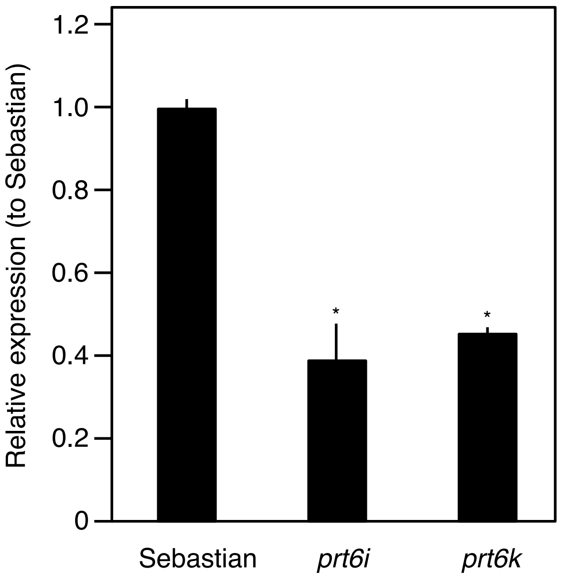


**Supplementary Figure 6**. qRT-PCR analysis of the influence of the *HvPRT6* mutant alleles *prt6k* and *prt6i* on *HvPRT6* gene expression relative to the WT (Sebastian). In each case error bars represent standard deviation of the mean of three independent experiments. *P<0.05.
